# Cardiac Meets Skeletal: What’s New in Microfluidic Models for Muscle Tissue Engineering

**DOI:** 10.3390/molecules21091128

**Published:** 2016-08-26

**Authors:** Roberta Visone, Mara Gilardi, Anna Marsano, Marco Rasponi, Simone Bersini, Matteo Moretti

**Affiliations:** 1 Department of Electronics, Information and Bioengineering, Politecnico Di Milano, Milano 20133, Italy; roberta.visone@polimi.it (R.V.); marco.rasponi@polimi.it (M.R.); 2 Cell and Tissue Engineering Lab, IRCCS Istituto Ortopedico Galeazzi, Milano 20161, Italy; mara.gilardi@grupposandonato.it; 3 Department of Biotechnology and Biosciences, PhD School in Life Sciences, University of Milano-Bicocca, Milano 20126, Italy; 4 Departments of Surgery and Biomedicine, University Basel, University Hospital Basel, Basel 4065, Switzerland; anna.marsano@usb.ch; 5 Regenerative Medicine Technologies Lab, Ente Ospedaliero Cantonale, Lugano 6900, Switzerland; 6 Swiss Institute for Regenerative Medicine, Lugano 6900, Switzerland; 7 Cardiocentro Ticino, Lugano 6900, Switzerland

**Keywords:** microfluidic, in vitro 3D model, skeletal muscle, cardiac muscle, heart, organ-on-a-chip, electrical stimulation, mechanical stimulation

## Abstract

In the last few years microfluidics and microfabrication technique principles have been extensively exploited for biomedical applications. In this framework, organs-on-a-chip represent promising tools to reproduce key features of functional tissue units within microscale culture chambers. These systems offer the possibility to investigate the effects of biochemical, mechanical, and electrical stimulations, which are usually applied to enhance the functionality of the engineered tissues. Since the functionality of muscle tissues relies on the 3D organization and on the perfect coupling between electrochemical stimulation and mechanical contraction, great efforts have been devoted to generate biomimetic skeletal and cardiac systems to allow high-throughput pathophysiological studies and drug screening. This review critically analyzes microfluidic platforms that were designed for skeletal and cardiac muscle tissue engineering. Our aim is to highlight which specific features of the engineered systems promoted a typical reorganization of the engineered construct and to discuss how promising design solutions exploited for skeletal muscle models could be applied to improve cardiac tissue models and vice versa.

## 1. Introduction

Why is microfluidics so appealing? The trend of publications containing the word microfluidics has continuously grown during the past years with a 55% net increase from 2010 to 2015 [1]. Microfluidic technologies offer the possibility to manipulate small amounts of fluids reducing the sample volume and the costs for reagents, while offering more control over the spatio-temporal phenomena occurring within the system [2].

Since the introduction of soft-lithography techniques based on poly-dimethyl-siloxane (PDMS) [3] and the development of micro total analysis systems [4], microfluidics has gradually become cheaper, e.g., paper microfluidics have found several applications [5], and are simpler, e.g., without the need of external pumping systems [6].

Recently, a specific application of microfluidic technologies has led to the development of advanced in vitro cell culture platforms, so-called organs-on-a-chip. By recapitulating key features of the natural architecture and the multicellular arrangement of the native tissue, these platforms allow one to model and analyze at the molecular, cellular, and tissue level a wide range of interactions that traditional systems are not able to dissect [2]. These promising tools are, thus, able to reproduce the pathophysiology of functional tissue units within micrometer sized chambers, as already demonstrated for bone, liver, lung, kidney, and other organs [7].

In addition to the advantages offered by the microscale size, the presence of a 3D microenvironment allows to effectively mimic structure and function of in vivo-like tissues, overcoming limitations of conventional 2D culture systems. In particular, several efforts have been made to develop organs-on-a-chip mimicking the complexity of heart and skeletal muscle, whose functionality is based on the effective coupling between electrochemical stimulation and mechanical contraction. In this context, electrical stimulation has been reported to increase cardiomyocyte spreading and alignment [8] and induce differentiation of embryonic stem cells towards a cardiac phenotype [9], while both cardiomyocytes [10] and myoblasts [11] respond to modulations of the mechanical environment. Several miniaturized systems have been developed to replicate electrical and mechanical stimulation patterns to enhance the functionality of cardiac and skeletal muscle tissues. However, although the application of electrical stimuli is generally considered a standard requirement, amplitude, frequency, and duration of the stimuli vary among different studies. Several works have demonstrated that it is possible to guide the cellular re-organization by tuning the properties of the substrate in terms of surface micropatterning and material features [12], or by modifying shape and stiffness of the 3D structure around which the tissue is grown [13,14,15,16]. Furthermore, dynamic culture conditions have been shown to increase the quality of skeletal muscle myofibers in terms of size, nuclei density and force generation [17]. Microfluidic technologies have shown to combine and simultaneously apply electrical, mechanical, and fluid flow-induced stimuli, and analyze their synergistic effect while fully exploiting the advantages of microscale models. Despite systems integrating all these stimuli do not currently exist, recent advances have demonstrated the possibility to mechanically condition 3D cultured cardiomyocytes with uniaxial cyclic strain, electrically pace the tissue construct and efficiently deliver drugs through adjacent microchannels [18].

Since a growing body of literature is rapidly emerging, it is necessary to analyze the state of the art of existing organs-on-a-chip mimicking heart and skeletal muscle, and explore the challenges and future directions. This review critically summarizes microfluidic platforms for cardiac and skeletal muscle tissue engineering that were developed during the last five years, highlighting which system architectures promoted specific features of the engineered tissue, and suggesting future directions by emphasizing how specific design solutions exploited for the skeletal side of the muscle could be applied to the cardiac side and vice versa.

## 2. Muscle Tissues: Engine of the Body

**Figure 1:**
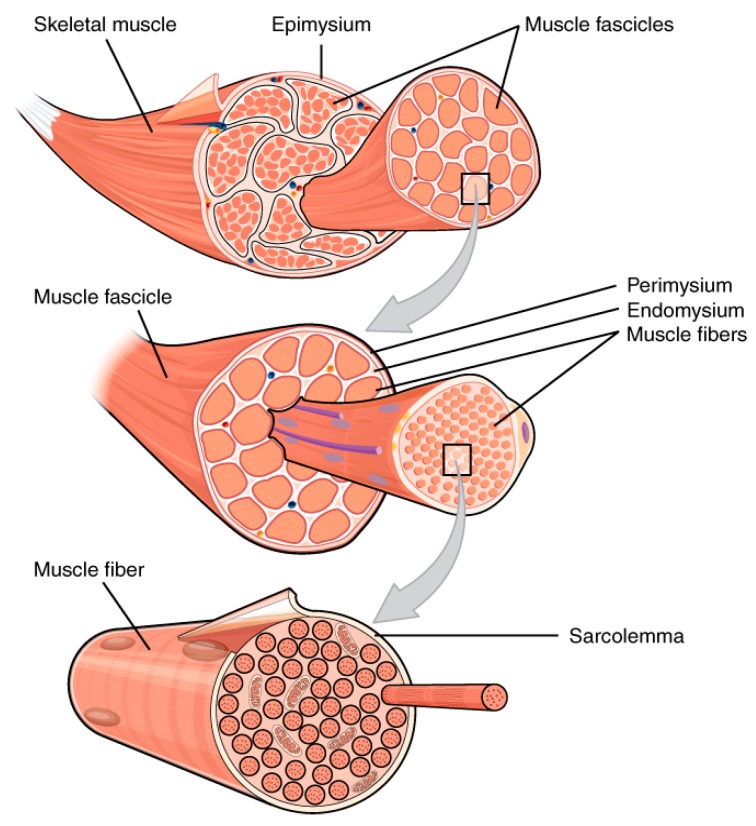
The hierarchical structure of the skeletal muscle. Reproduced from [19], Creative Commons Attribution License.

In the human body muscles develop strength and body work, and allow blood pumping through the beating heart. Based on their specialization, muscle tissues may be classified into three types: skeletal, cardiac, and smooth. From a structural point of view, the muscle is characterized by a hierarchical organization. Moving from the inside out, skeletal muscle is composed by myofilaments, sarcomeres, myofibrils, muscle fibers, and fascicles (Figure 1). Sarcomeres, the skeletal muscle contractile units, result from the assembly of thick (myosin based) and thin (actin based) myofilaments. The repetition of sarcomeres generates myofibrils, which assemble into myofibers, long cylindrical multinucleated cells. Muscle fibers are the basic unit of muscles and organize into fascicles, which are surrounded by a network of blood vessels and nerve fibers.

**Figure 2:**
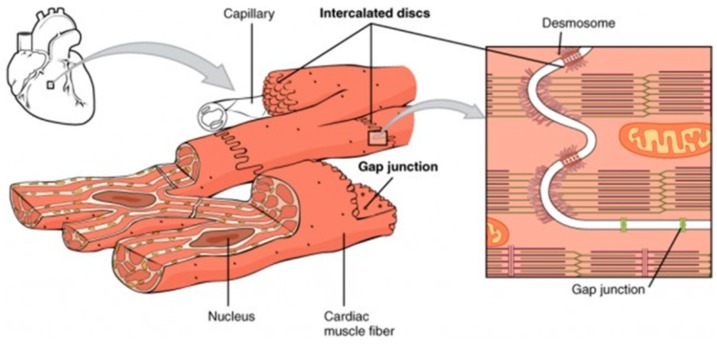
Structure of the myocardium functional unit. Reproduced from [30], Creative Commons Attribution License.

The myocardial structure presents similarities with the skeletal muscle [20] (Figure 2). Myosin and actin myofilament arrangement builds up the sarcomere, which is delimited by the Z lines. The serial repetition of sarcomeres forms the myofibrils, while multiple myofibrils compose the striated muscle cells, i.e., the muscle fibers that organize in anisotropic structures elongating and aligning on the extracellular matrix (ECM) [21]. Cardiomyocytes connect through specialized junctional complexes, the intercalated disks, characterized by an involuntary control system. The disks optimize the transmission of mechanical forces and provide electrical coupling thanks to the gap junction channels. This ionic continuity is fundamental for cellular electro-mechanical activation and allows cardiac muscle fibers to work together as a functional syncytium [22]. In addition to the fibrous structure, ventricular cardiomyocytes can also organize in laminar structures (4–6 fibers each) separated by connective tissue [23]. Despite similarities between the structures of skeletal and cardiac muscles, the cardiac tissue maintains its specific properties. Cardiomyocytes are mono-nucleated cells with the nucleus in the central part of the fiber. They present bigger myofibrils and a less organized sarcoplasmic reticulum. Lastly, the innervation system can just modulate and not generate the heart beat because cardiac muscle fibers contract spontaneously with an intrinsic rhythm given by pacemaker cells in the sinoatrial node.

Many problems can affect skeletal muscles and cardiac tissues. Skeletal muscle pathologies include genetic disorders such as muscular dystrophy [24], inflammation conditions such as myositis, and nerve diseases. These disorders could be responsible of pain and weakness and in the end paralysis and death. Concerning the heart, the main pathologies leading to the organ failure are related to ischemic, hypertensive and inflammatory diseases that are responsible for mechanical or electrical myocardial dysfunctions [25,26].

Considering the aim of this review, it is fundamental to compare morphological differences and similarities between skeletal and cardiac muscle in order to design customized in vitro systems for their study. For this reason, we would like to highlight the following parameters characterizing skeletal and cardiac muscle, such as presence of gap junctions, contraction regulation, source of calcium ions, and stimulation systems (nervous/pacemaker cells) (Table 1). Since muscles are characterized by a hierarchical organization, it is critical to analyze at different levels structure and function in order to define the mechanisms regulating these complex tissues [27]. Microfluidics offers the unique advantage to develop customized models whose complexity can be modulated according to the specific analytical need. It allows the generation of structures with well-defined shapes and positions, the precise cell and tissue manipulation, and the design of a highly structured 3D biomimetic microenvironment [28,29]. In this context, engineered tissue models of skeletal muscle and heart will provide a reliable tool to analyze physiology, disease development, molecular signaling, and drugs effects.

## 3. Microfluidic Approaches to Engineer Skeletal Muscle Tissue

Different strategies have so far been developed for the generation of functional skeletal muscle tissue constructs, including mixed in vitro/in vivo approaches which were employed to engineer muscle flaps to reconstruct large tissue defects [31]. Macroscale in vitro models were also designed to analyze the role of specific drugs on the muscle functionality [13]. As compared to these models, microfluidics offers the possibility to dissect the skeletal muscle function at different levels from the single cell behavior to the heterotypic interaction with other cell types, e.g., motoneurons, and to real-time monitor the cell behavior within biochemically- and biophysically-controlled microenvironments.

### 3.1. Cellular Level

**Figure 3:**
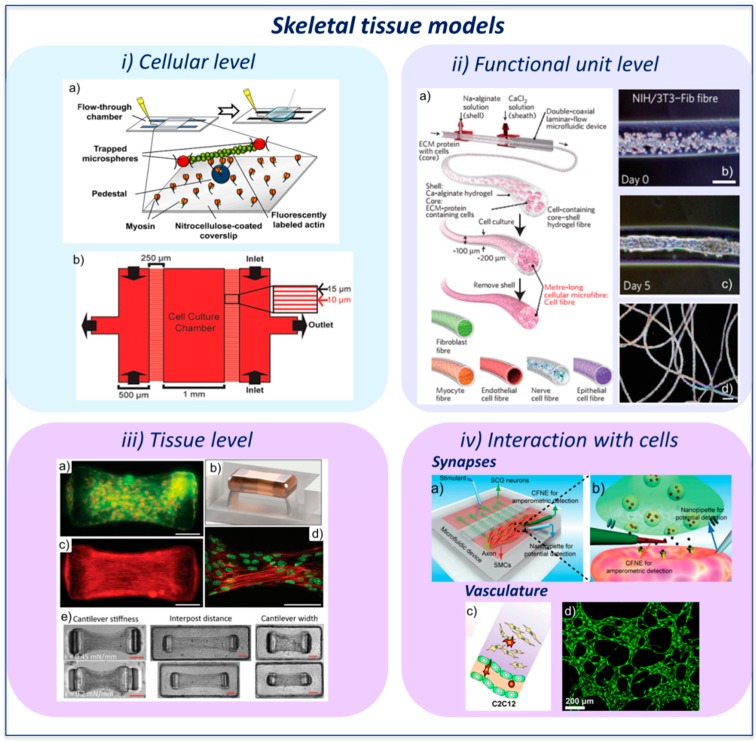
Microfluidic models of skeletal muscle. (**i-a**) Schematics and detail of a molecular mechanics assay chamber (left) and prototype of a microfluidic device (right) which allows to introduce chemicals without creating bulk flows compromising mechanics measurements. Reproduced by permission of the American Chemical Society [32]; (**i-b**) Microfluidic device for chemotaxis and chemokinesis assay. A microcapillary array separates the cell culture chamber from the outer channels which allow to introduce reagents and create a uniform concentration or a gradient in the chamber. Reproduced by permission of the Royal Society of Chemistry [33]; (**ii-a**) Generation of cell-laden microfibers containing different types of cells with a double coaxial microfluidic device; (**ii-b**) NIH/3T3 cells encapsulated within fibrin hydrogel at day 0 and (**ii-c**) cell fiber at day 5. Scale bar: 100 μm; (**ii-d**) Acid-solubilized type-I collagen fiber containing NIH/3T3 cells at day 4 post-seeding. Scale bar 500 μm. Reproduced by permission of the Nature Publishing Group [34]; (**iii-a**) Skeletal muscle microtissue immunofluorescence (cell membrane bound green fluorescent protein (GFP) signal and nuclear staining (red)) showing uniform cell distribution; (**iii-b**) CAD modeling of a skeletal muscle microtissue; (**iii-c**) F-actin (red) staining demonstrating myoblast alignment after three days of culture and (**iii-d**) actin filament remodeling within multinucleated myotubes (nuclei are stained in green); (**iii-e**) Effect of structural (interpost distance, cantilever width) and mechanical (cantilever stiffness) parameters on the skeletal muscle microtissue. Scale bars: 100 μm. Reproduced by permission of the Royal Society of Chemistry [16]; (**iv-a**) Schematic of the engineered neuromuscular junction within a microfluidic device; (**iv-b**) schematic of the insertion of a carbon fiber nanoelectrode (CFNE) within the synapse for amperometric measurements and of a glass nanopipette inside a muscle cell (red) to record the post-synaptic potential. SCG: superior cervical ganglion. Reproduced by permission of Wiley-VCH [35]; (**iv-c**) simplified sketch showing a vascularized muscle-mimicking microenvironment for cancer cell extravasation studies. Endothelial cells (green), cancer cells (red) and muscle cells (yellow); and (**iv-d**) GFP-labelled microvascular network embedded within organ-specific matrix in a microfluidic device. Reproduced by permission from Jeon, J.S.; et al. [36].

In the field of sub-cellular analyses of skeletal muscle myofiber regulation, the actin-myosin interaction represents a key aspect to understand the muscle contraction. The common setup for the protein-protein interaction study is the flow-through chamber in which buffers and proteins are sequentially perfused. These molecular analyses are extremely sensitive and the addition of reagents that could introduce shear stress would invalidate the measurements. Roman et al. developed a microfluidic device that allows to control chemical flows in a flow-through chamber while avoiding the creation of any bulk flow that could disturb the molecular mechanics analyses (Figure 3(i-a)) [32].

Introducing analyses at the cellular level to study muscle fibers, microfluidic approaches were employed to study myoblast migration and their response to molecular factors and ECM stimuli. 2D cultures or transwell assays represent the traditional choices to assess the effect of different stimuli, such as signaling molecules, on cell migration. A classic example is the culture flask or Petri dish in which the medium supplemented with chemo-attractant molecules is added to the cell layer. Alternatively, cells are seeded on a porous membrane, which is placed in a multi-well containing the chemo-attractant. Furthermore, it is often hard to investigate, through in vivo models, the migratory behavior of single cells. The reason of this is the lack of high-throughput and high spatio-temporal resolution tools allowing analyses at the single cell level. As Ferreira et al. demonstrated, traditional transwell assays fail to produce stable gradient concentrations, which are required to quantify chemotaxis phenomena [33]. The authors used a microfluidic approach that allows longitudinal, quantitative, single-cell migration analyses of primary human myoblasts from patients (Figure 3(i-b)). They demonstrated that changes in ECM, as a result of aging and disease, could impact the regeneration of muscle fibers, modifying migration and proliferation. Furthermore, they showed that basic fibroblast growth factor (bFGF) main role is chemokinesis rather than chemotaxis, and that other growth factors, either alone or in combination with bFGF, are needed to initiate directed cell migration, driving tissue healing after muscle injury. In this case, microfluidics not only enable distinguishing among chemoproliferation, chemokinesis, and chemotaxis, but also to analyze the contribution of ECM proteins (e.g., fibronectin, collagen, and laminin) in the definition of those behaviors.

### 3.2. Functional Unit Level

Engineered cell-laden fiber-shaped constructs represent a key example of building block to assemble higher level structures, mimicking the hierarchical architecture of the organs in the human body. Onoe et al. employed microfluidic techniques to develop a fiber-based strategy to create 3D complex tissues (Figure 3(ii-a)) in vitro. Particularly, cell-containing ECM-protein/Ca-alginate core–shell microfibers were assembled through a double-coaxial microfluidic device. The cells contained within the tubular ECM-protein (Figure 3(ii-b)) gradually dispersed creating fiber-shaped microfibers (Figure 3(ii-c,ii-d)) that mimic basic functions of living tissues [34]. The authors used ten different cell types, such as myocyte and cardiomyocyte, endothelial cells, fibroblasts, nerve cells, and epithelial cells. Furthermore, cell fibers were fabricated using three types of ECM protein with different compositions and mechanical stiffness consisting in pepsin-solubilized type-I collagen, acid-solubilized type-I collagen and fibrin. It is interesting to note that not all the cell types were able to generate fibers in the different ECMs. More in detail, the authors demonstrated that the success of fiber formation depends on the combination of cell types and ECM proteins. In particular, pepsin-solubilized type-I collagen core failed to form cell fibers from fibroblasts, endothelial cells, and myocytes. Since it is known that these cells need a stiffer scaffold to adhere and stretch, the reason why fibers did not form could be that the ECM was not stiff enough to promote cell-cell connections. The authors also showed that assembling fibers by means of weaving and reeling allows to reach macroscopic cellular structures with different spatial design. This approach could be applied to a broad range of tissues, including cardiac and skeletal muscle. Indeed, since the muscle could be assimilated to a cluster of fibers, its analysis at the fiber level is characterized by high biological relevance. Unfortunately, traditional 2D culture assays do not allow mimicking the 3D bundle structure of the fiber.

Concerning the generation of functional units of the skeletal muscle, meant as a single muscle fiber in microfluidic systems, no other study was recently reported in the literature. For this reason we believe that more efforts could be done in this field, since the functional unit represents the building block of muscle tissues.

### 3.3. Tissue Level

The primary function of skeletal muscle is contraction and the generation of mechanical force. The force generated by an engineered tissue varies depending on multiple conditions, including displacement, creep, stress relaxation, load impedance, fatigue, and pre-stress. In addition, the stimulation setup can affect muscle contraction. For this reason, it is fundamental to develop in vitro 3D models of muscle contraction that allow to investigate the effects of different stimulation patterns including chemical, optical, and electrical.

Shimizu et al. developed a 3D contractile skeletal muscle tissue cultured in a microfluidic channel. Immunofluorescence analysis on the mouse cell line C2C12 confirmed the formation of myotubes one week after seeding in type-1 collagen, and tissue contraction upon application of electrical stimulation [37]. Electrical and chemical stimulation might lead to cell stress due to changes of the culture parameters (e.g., pH). To overcome these issues and to increase the spatial and temporal resolution, Sakar et al. designed and developed a novel method to optically stimulate the cells by using skeletal myoblasts expressing channel rhodopsin-2, a light-activated cation channel. Using precise optical stimulation the generated myotubes were activated individually and as a group. More in detail, once introduced in the device, self-assembled longitudinally-aligned and differentiated myofibers were generated from seeded cells. After that, the authors characterized the contractility of the tissue upon exposure to brief light pulses and by monitoring the deformations of the elastic microposts composing the device. Overall, this system, called “skeletal muscle on a chip”, represents a reliable model which allows performing contraction measurements upon application of an external stimulus (Figure 3iii) [16].

The skeletal muscle force is generally measured by confining the fiber terminations at a fixed position relative to each other. However, this approach does not allow real-time measurements of the force in response to an external applied displacement [38]. Despite not properly belonging to the microfluidic category, it is worth mentioning the work by Neal et al. who developed a reliable tool to analyze in a quantitative way, real-time, the displacement-dependent muscle behavior. In this study, the authors designed a platform to quantify the mechanical output of engineered muscle tissues and compared the effect of electrical and optical stimulation [14].

### 3.4. Interaction with Other Cells

Muscle contraction is strictly coupled with the excitation pattern of the motoneurons. Particularly, the site of signal transmission is called neuromuscular junction (NMJ). The motoneuron work and the neuromuscular junction function involve cycles of necrosis and regeneration that, with time in the pathological condition, could lead to serious illness, such as amyotrophic lateral sclerosis (ALS), spinal muscular atrophy (SMA), and myasthenia gravis. ALS is a disease involving the death of neurons that control voluntary muscles, SMA induces physical strength loose affecting the motor nerve cells in the spinal cord, and myasthenia gravis is related to a deficit in the acetylcholine function at the neuromuscular junction level. Modeling these degenerative processes is critical to understand the mechanisms underlying muscle contraction diseases. However, 2D cultures do not allow to reliably study these phenomena which involve the coupling of different tissues. Ionescu et al. developed a method to study the mechanisms of NMJ formation, maintenance and disruption in a 3D environment. Particularly, green fluorescent protein (GFP) labelled motoneurons from mice were introduced in one side of a microfluidic device and myotubes were plated on the other side. The motoneurons innervated the muscle fibers and formed functional NMJs. Furthermore, the described system was dedicated to the analysis of the NMJ biology thanks to the possibility to independently manipulate and observe the pre- and post-synaptic cell compartments [39]. Additionally, Li et al. developed a model allowing the detection of individual neurotransmitter release events inside functional synapses between superior cervical ganglion neurons and their effector smooth muscle cells (Figure 3(iv-a)). The measurements were performed with carbon fiber nanoelectrodes and post-synaptic potential was recorded using glass nanopipette electrodes (Figure 3(iv-b)). Despite this work not being performed with skeletal muscle cells, the described approach permits to monitor the synaptic transmission and could be translated to skeletal muscle studies [35]. By means of rat primary cells, Southam et al. generated a microfluidic model of motoneuron growth reproducing the spatial structure between motoneurons and glial/muscle cells. This model allowed the analysis of the behavior of neuromuscular connections between axons and skeletal muscle cells [40].

Considering the muscle tissue physiology, vascularization has a central role for the generation of advanced in vitro muscle models. For these reasons several strategies have been developed to obtain complex vascular networks, such as vasculogenesis-based techniques [41] and bioprinting strategies [42]. It is of basic importance to study the interactions between vessels and tissues. In this context, a vascularized microfluidic model of skeletal muscle was developed to study how the presence of muscle cells can affect endothelial permeability (Figure 3(iv-c,iv-d)) [36].

These models could pave the way for a new generation of microfluidic systems embedding vascularized and innervated muscle constructs better recapitulating different aspects of muscle tissues. To reach this milestone, researchers should consider physiological characteristics of in vivo muscle fibers. In particular, since the high metabolic activity of muscles, it is important to consider oxygen and nutrient consumption to develop reliable engineered models [43]. Finally, the application of microfluidics to skeletal muscle tissue engineering has the potential to promote the design of drug screening and toxicological tests analyzing the effects of specific molecules in a more physiological environment.

## 4. Cardiac Microfluidic Models

Different strategies involving tissue engineering methods have been so far proposed to generate in vitro cardiac tissue models, aiming at reproducing myocardium intrinsic complexity and organization [44] to study its physiological or pathological conditions [45]. Among others, promising strategies have exploited microfluidic platforms to reproduce the miniaturized cell culture environments. These systems not only offer the possibility to precisely regulate the local cellular microenvironment to study the influence of the heart tissue “niche”, but also allow to perform studies in a high-throughput way and in a cost effective manner [46]. Here, we present different microfluidic platforms, with different complexity levels, each one designed to recapitulate a particular feature of the myocardium organization. Investigating how each characteristic affects the development and behavior of cardiac constructs can help in generating more relevant cardiac tissue models aiming at performing reliable drug screening tests or developmental biology studies [47].

### 4.1. Cellular Level

**Figure 4:**
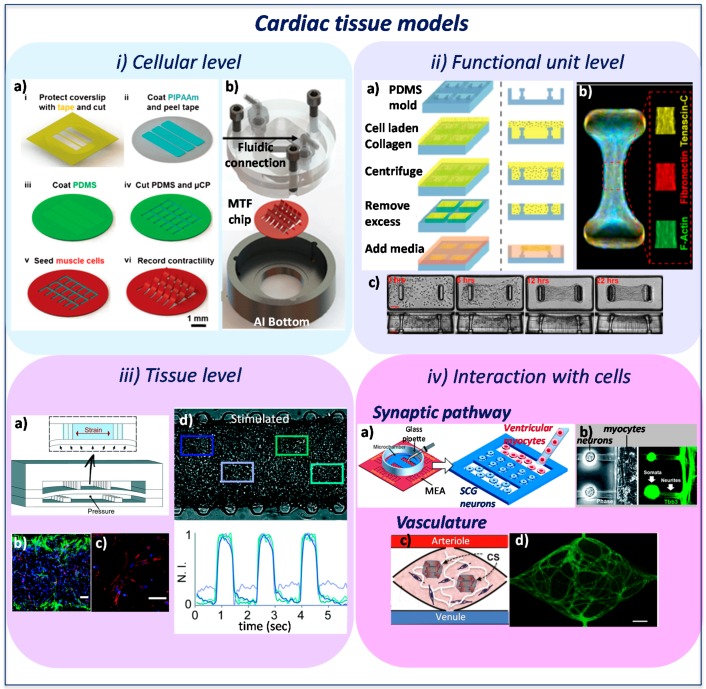
Platforms designed to create in vitro cardiac tissue models to investigate different features of myocardial organization. At cellular level, the muscular thin film (MTF) [49] used to investigate the cardiac functional contractility related to the sarcomere organization: (**i-a**) schematic step-by-step representation of MTF fabrication processes and (**i-b**) the fluidic device assembly. At the functional unit level, the microfabricated tissue gauges (µTUGs) [51] exploited to study the functional contractility related to cell alignment and reorganization: (**ii-a**) process flow of µTUGs creation; (**ii-b**) immunofluorescence of ECM and cytoskeletal proteins in microtissues and (**ii-c**) time course of a contracting cardiac microtissue. At tissue level, the microengineered cardiac tissue (µECTs) [18] showing physiological beating in response to physiological mechanical stimulation: (**iii-a**) design of the 3D heart-on-chip microdevice used to impose a strain to the tissue by pressurizing the bottom compartment; (**iii-b**) immunofluorescence of cardiac troponin I (green) and connexin 43 (red) and (**iii-c**) sarcomeric-α-actinin (red) after five days in culture (scale bar 100 µm); (**iii-d**) evaluation of the contraction period in four different areas of the constructs showing the beating synchronicity reached by tissues. To study the interaction with other cells, platforms to reproduce the synaptic pathway [52] (**iv-a**,**iv-b**) and the cardiac vasculature [53] (**iv-c**,**iv-d**): (**iv-a**) seeding procedure of PDMS microchambers on a micro electrode array (MEA) surface to separately co-culture neurons and myocytes; (**iv-b**) fluorescence immunostaining showing the neurite extending in the myocyte compartment; (**iv-c**) scheme of designed platform to create vasculature around cardiac muscle spheroid (CS) and (**iv-d**) fluorescence microscopy of CD31-stained (green) vessels within the microtissues (scale bar 200 µm).

The cardiac ventricle wall exhibits locally laminar anisotropic structures. Parker and co-workers designed a microfluidic platform where 2D muscular thin films (MTFs), formed by engineered anisotropic muscular tissue, are grown on top of fibronectin-patterned flexible elastomeric cantilevers (Figure 4(i-a)) [48]. Contractile functional data of these anisotropic cellular monolayers were assessed and correlated with sarcomere organization (sarcomeric α-actinin staining). The authors demonstrated that a high degree of alignment corresponds to higher systolic and diastolic stresses. Furthermore, improving this platform by integrating a fluid flow control system and a couple of platinum electrodes, allowed them to perform more complete contractility tests, investigating MTF behavior under electrical stimulation (Figure 4(i-b)) [49]. The platform was positively tested for drug screening purposes, verifying that the engineered cardiac microtissues undergoing isoproterenol (β adrenergic agonist) treatments generated higher contractile stresses. This work successfully demonstrated that cardiomyocytes can generate relevant contractile forces, within the range measured in isolated muscles, when cells align in 2D structures and the constructs are properly stimulated [50].

Other systems were developed to evaluate more in detail the electrical conductivity of the generated tissue. Indeed, in the heart the electrical conduction begins with an action potential generated by the cardiac pacemaker cells [54], while the unidirectional propagation of the electrical pulse along the aligned muscle fiber is ensured by end to end intracellular junction proteins [55]. Based on the previously evidenced concept that structure determines function, Ma and colleagues [56] exploited lithographic techniques to microfabricate multiple microwells on top of micro electrode array (MEA) surfaces. Within the wells, cardiomyocytes aligned to generate in vivo-like cardiac muscle fibers showing fast conduction velocity and action potential similar to those found in adult mouse myocardium [57]. Furthermore, the authors investigated the capability of different cell sources (cardiomyocytes, stem cells, fibroblasts) to couple with already-formed cardiac fibers through the formation of conductive cell bridges [56]. Briefly, a portion of each cardiac fiber was removed to deposit a controlled number of different cells through a laser-patterning technique. Among the cell types tested, cardiomyocytes showed an efficient integration with the previously formed fibers already within 12 h, the mesenchymal stem cells integrated in five days, while fibroblasts were unable to maintain a stable coupling with the cardiomyocytes. These promising results are related to the capabilities of different cell sources to form new gap junctions with the hosting tissue and can give new hints to solve problems related to the poor integration between cardiac tissue constructs once implanted in the native tissue.

A combination of these previously described approaches was exploited by Stancescu and co-workers [58]. The authors developed an in vitro parallel environment integrating a 2D culture system with a biomicroelectromechanical system (BioMEMS) to culture human stem cell derived cardiomyocytes. The 2D system consists of a MEA surface patterned with fibronectin protein by means of surface modification and photolithographic techniques. The BioMEMS is a cantilever-based force measurement system composed by a fibronectin coated silicon cantilever and an atomic force microscopy (AFM). The combination of these two in vitro test systems enabled to measure defined parameters that have a high predictive value for biologically relevant cardiac functions like conduction velocity, action potential length and force generation. This approach highlighted the rising needs to develop new platforms that, aside from allowing cell culture and re-organization, integrate different systems to directly investigate the properties of the forming construct.

To better investigate the electrical properties of cardiac cells, other groups focused on a key functional feature of cardiomyocytes: the calcium handling machinery. Indeed, Martewicz et al. specifically designed a microfluidic system able to perform on line investigation of intracellular cardiomyocyte calcium concentration under highly controllable fast oxygen concentration dynamics [59]. They demonstrated that during the early phase of a hypoxic event, the cardiomyocyte intracellular Ca^2+^ dynamic is impaired and can be recovered just for hypoxic levels below the 5% O_2_ partial pressure. This functional alteration shows an adaptive cardiopreservation mechanism activated during ischemic events.

Overall, these models recapitulate the most important aspects to be considered (mechanical and electrical performances) for analyzing the maturation and functionality of 2D cultured cardiomyocytes.

### 4.2. Functional Unit Level

To develop more realistic 3D biomimetic tissues, microfluidic devices were designed to culture under continuous perfusion, self-assembled aggregates of stem cells. For example, Bergstrom generated cardiac bodies (CBs) and captured them in “niches” arranged along a perfusion channel within a microfluidic device [60]. The CBs, clusters of human induced pluripotent stem cells derived cardiomyocytes (hiPS-CM), showed similarities with the native human myocardium. Indeed, the cells organized in longitudinally-aligned cardiomyocytes, exhibited well-developed sarcomeres and expressed Nkx2.5 and Cx43 cardiac markers [61]. Taking advantage from the possibility to establish a controlled environment inside each niche, the beating frequency of CBs treated with different therapeutic drugs was measured using an ordinary microscope. The non-invasive nature of the beating frequency measurement and the high number of CBs in each channel make the platform appealing to perform drug screening on microtissues. In addition to the beating frequency of the cardiac model, the force generated by the tissue should also be assessed to better characterize the maturation of the developed constructs, as demonstrated by Park and colleagues. Huesbsh and co-workers described an approach that combines the engineered heart muscle (EHM) technique at the macroscale [62,63,64] with the cardiospheres formation [65], creating micro-heart muscle (μHM) arrays [66]. Each µHM is formed by less than 10,000 iPS-CMs and iPS-fibroblasts seeded in different ratios into molds presenting high aspect ratio and an optimized dogbone shape. The cells elongated and achieved uniaxial alignment and robust sarcomere assembly already after three days. These μHMs showed physiologically-relevant drug responsiveness and exhibited reproducible Starling responses, as demonstrated by measurement on a standard twitch force measurement apparatus.

In order to evaluate the functionality of the cardiac tissue constructs, different microscale approaches were exploited to guide the formation of cell-laden hydrogels in specific zones and allow in situ real time measurements. Aung et al. developed 3D cardiac perfused cell-laden hydrogels within a microfluidic device with the possibility to quantify the contractile stress of microtissue in real-time and through in situ measurements [67]. Exploiting a 3D patterning technology, the authors guided the photo-crosslinking of cardiomyocyte-laden methacrylated gelatin (GelMA) hydrogel in precise points. The obtained spatially-distributed cardiac microtissues were sandwiched between two polyacrylamide (PA) hydrogels embedding fluorescent beads that were used as “stress sensors”. The contractile stresses generated by the beating cardiac cells were thus calculated from particle displacements, through a finite element model (FEM). The engineered cardiac tissue exhibited increased stress amplitude and beating frequency in response to exogenous single dose epinephrine, but the generated contractile stress of constructs was low, likely due to poorly organized myofibers. This was probably related to the fact that inside the platform none of the structures guided the cells to reorganize into anisotropic structures.

Since different studies highlighted important connections between cell mechanics and cell phenotype [68,69], as well as between cell organization and performances [27], different platforms were designed to drive the self-assembly of fiber-shaped, cell-laden hydrogels which are anchored and aligned along the axis of pairs of posts. Legant and colleagues exploited the microelectromechanical systems (MEMS) technology and developed microfabricated tissue gauges (µTUGs) by embedding cells within 3D matrices and anchoring them around microcantilevers (Figure 4(ii-a)) [51]. These cantilevers simultaneously constrained the hydrogel remodeling and reported, in real-time, the forces generated by 3D constructs. The cantilevers’ mechanical stiffness and hydrogel matrix modulus altered the contractile force of the construct and the deposition matrix proteins, suggesting their influence on 3D tissue organization (Figure 4(ii-b,ii-c)). This technology was also fully exploited in the work of Boudou et al. [70], where the T-shaped MEMS were used to generate arrays of cardiac microtissues (CMTs) within collagen/fibrin hydrogels [71]. By increasing the spring constant of the cantilevers the system rigidity increased, leading to a superior auxotonic load, which resulted into more mature sarcomeric structure formation, cell alignment and re-organization. On the other side, the increase in hydrogel stiffness impacted on the ability of the cells to reorganize and remodel the matrix, resulting in a poor alignment of the cells and a lower efficiency of the cardiac tissue. Furthermore, given that electrical stimulation improves structural and functional properties of engineered myocardium [72,73], the CMTs were electrically stimulated and showed better compaction of the matrix and faster alignment of the cells, improving the cell coupling and increasing the positive effect of auxotonic loads. Additionally, CMTs were demonstrated to be suitable for drug screening tests, exhibiting a dose-dependent variation of contractility and beating frequency after isoproterenol and digoxin administration. Exploiting this technique, Thavandiran and co-workers presented an integrated computational and experimental strategy to guide the design of functional 3D human pluripotent stem cell-derived cardiomyocytes (hPSC-CM) to form cardiac microwires (CMWs) and to establish a tachycardic model of arrhythmogenesis [74]. A finite element model (FEM) predicted the dynamic reorganization and contractility of cytoskeleton in relation to the geometrical cues of the environment, elucidating the relationship between local stress state of the fiber and the sarcomere formation. Furthermore, pacing the self-assembly 3D tissues with point stimulation electrodes promoted the expression of cardiac maturation-associated genes and in vivo-like electrical signal propagation [75,76].

All together, these platforms evidenced the importance to develop new systems with suitable structural and mechanical properties, promoting the generation of functional tissues.

### 4.3. Tissue Level

Even if some specific cues of cardiac tissue were gained in the previously described systems, the complete set of microenvironmental parameters typical of the myocardial niche was not recapitulated. With this perspective, Mathur and colleagues developed a cardiac microphysiological system (MPS) to recapitulate the minimal organoid structure of the human myocardium. The authors designed a microfluidic device where the central channel, with its biomimetic dimension, forced the self-organization of human induced pluripotent stem cell derived cardiomyocytes (hiPSC-CMs) into aligned 3D microtissues [77,78]. Nutrients were continuously exchanged from the two lateral channels through 2 µm-wide microchannels that mimicked the endothelial barrier, protecting tissue from shear stress and controlling diffusive transport of molecules. After 24 h from seeding, hiPSC-CMs formed a 3D cardiac tissue showing spontaneous physiological beating and, after seven days, the beats became aligned along the long axis of the MPS. The constructs were also tested with multiple drugs at different concentrations and the results showed that IC_50_ and EC_50_ values were more consistent with data at tissue-scale level compared to cellular-scale studies. These results demonstrated that the platform allowed cells to reach a minimal physiological level of reorganization, even without mechanical and electrical stimuli. Exploiting the recent organ-on-chip technology [7] Marsano et al. reported a new method to generate mature and highly functional 3D micro-engineered cardiac tissue (µECTs) within a microfluidic device from neonatal rat cardiomyocytes or hiPSC-CM [18]. With the aim of mimicking the native cardiac milieu, the authors designed an innovative heart-on-a-chip platform (Figure 4(iii-a)). The device was able to: (i) generate 3D mature microtissues with well-defined geometries from cell-laden hydrogels; (ii) mechanically stimulate the µECTs with the application of physiological cyclic uniaxial strains; and (iii) efficiently deliver chemicals and drugs for the biochemical environment conditioning. Altogether, these particular cues led to generate mature µECTs exhibiting superior micro- and ultra-structures, with enhanced mechanical and electrical coupling between neighboring cells. Indeed, within stimulated µECTs cells remodeled the embedding vehicle by substituting it with new deposited ECM and acquired a more complex organization of sarcomeres, intercalated disks and gap junctions (Figure 4(iii-b,iii-c)). The enhanced electrical performance was confirmed by the synchronous contractile activity of the µECT and by the low excitation threshold (ET) and high contraction amplitude reported during electrical pacing tests (Figure 4(iii-d)). Following a similar approach, Nunes et al. developed an interesting microfabricated bioreactor aiming at reproducing the natural organization of cardiomyocytes into spatially well-oriented cardiac bundles with supporting vasculature, evolving the system from their previously described biological wire [79]. Indeed, the maturation of cardiomyocytes from iPSCs in the original platform was achieved with electrical stimulation and reproducing the complex cardiac architecture, but nothing mimicked the native physiological myocardial mass transfer. In the more recent platform [80], perfused biowires were generated from neonatal rat cardiomyocytes and human embryonic stem cells embedded in collagen hydrogel and seeded on a polytetrafluoroethylene tubing template with dimensions resembling the post-capillary venules [81]. The circular template provided a topographical structure to guide cell elongation and alignment, reproducing the anisotropic structure of native myocardium. The electrical stimulation enhanced the tissue stiffness under a parallel electric field imposition by increasing the cardiomyocyte contractile apparatus organization (cardiac troponin T oriented along wire axis). In addition, it improved the maturation of hPSC-CM that showed mature myofibril structures and enhanced electrical properties. The perfusion system coupled with a parallel electrical stimulation improved the electrical properties of the biowires, as confirmed by the lower ET and the higher maximum capture rate (MCR), as well as from the improved cell alignment and interconnectivity.

All of these platforms showed well how mechanical, electrical, and chemical stimulations can improve the properties of the cultured constructs. However, as previously shown in this review, more efforts should be performed to incorporate systems allowing real-time analyses of the tissue.

### 4.4. Interaction with Other Cells

The heart contains two types of cells: excitable and non-excitable. The first ones are involved in the electrical signal propagation, while the others contract following their pacing [82]. Cardiomyocytes in vivo interact with fibroblasts, endothelial cells, and neurons. This cellular dynamic communication takes place through chemical, mechanical and electrical signals, and finely regulates heart function and development [83]. In particular, sympathetic neurons are responsible of regulating the heartbeat rate, the velocity of conduction, and the myocardial mechanism of contraction/relaxation. Takeuchi et al. developed different systems to investigate the effect of small cell networks formed by sympathetic neurons connected to cardiomyocytes [52,84]. The platforms are composed by MEA surfaces and PDMS-based culture chambers that allow to compartmentally co-culture neurons and cardiomyocytes or iPSC-CMs, achieving the functional synaptic pathway between the two cell populations (Figure 4(iv-a,iv-b)). In these platforms it is possible to investigate the cardiac modulation (changes in the construct beating rate) exerted by stimulating (electrical stimulation) the sympathetic nervous system. Another important characteristic of the myocardium is the high density of capillaries [85], so new biomimetic systems should include a relevant vascular supply. This approach was followed by Moya et al., who designed an advanced 3D micro-physiological platform in which the perfused microvessels developed and integrated with 3D contractile cardiac organoids from iPSC-CMs inserted in a microfluidic platform (Figure 4(iv-c,iv-d)) [53]. This strategy leads to a better mimicry of the natural tissue environment with the advantage of delivering in a physiological way nutrients and drugs to the tissue.

## 5. Discussion

We have highlighted some of the similarities between cardiac and skeletal muscle tissues and we have discussed different approaches that were used to analyze their features at different levels, from actin-myosin interactions to the contraction of the whole tissue.

In the field of cardiac tissue engineering two interesting strategies, aiming at studying in a high-throughput way the effect of different drugs, were developed. Cardiac 2D muscle strips [49] allowed the measurement of the stress generated by the constructs, while the approach based on cardiac bodies [60] permitted to analyze the spontaneous beating frequency of 3D tissue constructs. These strategies could be translated to skeletal muscle models to study the pathophysiology of the tissue.

Combining the advantages evidenced by Bergstrom et al. [60] in performing high-throughput drug screening with the force readout of the heart-on-a-chip designed by Agarwal and co-authors [49], it would be possible to develop novel and more relevant muscle models. Concerning the development of more realistic models, noticeable is the work by Das et al. who used serum-free media to perform more consistent studies. In fact, the authors developed one of the first protocols to differentiate fetal rat skeletal muscle cells and generate aligned myotubes on a microcantilever [86]. Two key aspects of this method are represented by the use of serum-free medium with a special cocktail of growth factors including brain-derived neurotrophic factor and glial-derived neurotrophic factor, and the surface coating treatment of the microcantilever with a cell growth promoting silane substrate.

Furthermore, although not belonging to the last five years of literature, and despite it employed rat and chicken embryo fibroblasts, we have included the work by Marquez et al. since the approach seemed promising to be translated in microfluidic devices for drug screening tests measuring the contractility of muscle fibers [87]. Particularly, the authors developed a system aimed at characterizing the mechanical properties of hydrogel tissue constructs embedding cells treated with different compounds which modulate the contractile activity. These constructs were grown in suitably-designed chambers and an automated system was used to measure the resulting forces induced by pretension. The authors demonstrated the effectiveness of the system to react to different stimuli. This approach could be implemented in microfluidic systems for drug screening tests in order to couple the high throughput nature of microscale models with the reliability of data obtained through robotic systems.

Microfluidic approaches were developed to study chemotaxis phenomena and the effect of ECM proteins on cellular migration [33] in tissue muscle models. This approach could be potentially used to investigate the migration of skeletal muscle satellite cells in the framework of wound healing and muscle regeneration. Furthermore, it would be of great interest to perform similar studies in a cardiac model contest, for example to study the recruitment of progenitor cells of the cardiac niche during inflammation or infarction and to identify new drug candidates helping in treating heart pathologies. In this context, it is of great interest the microfluidic platform designed to study the adaptive capacity of different cell candidates (cardiomyocytes, fibroblasts, and stem cells) to re-establish the functional electrical conduction of a truncated cardiac fiber [56]. Trying to elucidate this particular mechanism can give novel hints to develop new cell therapies to treat a damaged heart muscle and recover from infarction. Similar approaches are still missing for the study of the skeletal muscles and could have a great impact for the treatment of diseases which affect the transmission of the electrical pulse and consequently impair muscle contraction. Then, investigating the construct performances can be of great usefulness. In this field, Smith et al. employed a simple cantilever system capable of electrically stimulating and measuring the contractile activity of individual human muscle myotubes in a non-invasive manner [88]. A similar and interesting approach was also exploited by Stancescu and co-workers [58] who highlighted the importance to investigate the properties of the cardiac constructs by integrating electrodes to measure cardiomyocyte electrical activity and cantilevers to investigate mechanical properties of the generated tissues.

Despite being useful, all of these systems do not allow to fully recapitulate the architecture of the native muscle, since fiber-like constructs would better reproduce and mimic the hierarchical structure of this tissue.

In this framework, Onoe introduced the fiber-based strategy which could be applied theoretically to engineer functional building blocks that can be assembled with different methods to reconstruct complex tissues (i.e., neuronal, vascular, muscular) [34]. Furthermore, it is interesting to highlight that tuning the matrix composition and its mechanical properties could lead to different cell behaviors, e.g., diverse cell differentiation levels and fibers maturation. Other approaches were used to reproduce the basic functional unit of cardiac [51,66,70] and skeletal muscle [14] tissues, the bundles. Different authors indeed exploited microfabrication techniques to miniaturize in vitro models of aligned muscle fibers, based on the multiwell-plate analytics and the force contraction measurements optimized by Eschenhagen and Vandenburgh [64,89]. In fact, microfabricated molds containing posts and cantilevers were fully exploited to guide the spontaneous organization of cell-laden hydrogels (i.e., fibrin, collagen) to interchangeably form cardiac or skeletal muscle fibers. These particular elements can be used as passive components to both anchor the fiber during its development or to measure the stress generated from its contraction [16]. So far, no works are reported in which these pillars are exploited as active components to introduce physiological mechanical stimulation (uniaxial stretching instead of auxotonic load) to study the induction of muscular maturation in vitro [90,91].

Another interesting method that can substitute the bending posts technique to investigate the mechanical properties of the construct is based on fluorescent beads. These particles were successfully employed in cardiac models to monitor the stress generated from 3D engineered tissues and to gain information about the maturation stage of the construct [67]. This strategy could be easily translated to skeletal muscle studies.

Although not at the microscale and not in vitro, we would like to highlight the work by Cezar et al. who introduced the concept of differentiation and regeneration of the muscle fibers through mechanical compression in vivo. The mechanical compression could be inserted in microscale models as a stimulus conditioning the differentiation of both cardiac and skeletal muscle without the use of growth factors. Applying mechanotherapy may be an additive strategy to regenerate injured muscles in the patient treatment [92].

The electrical stimulation has been shown to induce the differentiation, enhance the functionality and promote the contraction of cardiac muscle [72,73] and skeletal muscle tissues [93]. Despite the major advancements of microscale systems, several crucial issues remain open. Above all, the choice of the electrode material, given that redox reactions could impair the integrity of the electrode and lead to the generation of toxic by-products. Moreover, it is difficult to embed the physical electrode in the microscale model due to its size and microfabrication limitations. To overcome this drawback, the most common approach relies on the metal deposition pattern technique which, however, allows to generate only planar electrodes. Recently, Pavesi et al. developed a new strategy to directly embed within microfluidic devices flexible PDMS and conductive particles to generate 3D electrodes and provide uniform electric fields [94,95]. In the end, the complex phenomena occurring at the electrode-electrolyte interface are difficult to predict. To avoid all of these problems, optogenetic approaches represent a suitable alternative to electrical stimulation, to induce contraction through the manipulation of cell membrane voltage [96,97,98]. Even if Neal et al. efficiently exploited this technique to stimulate the contraction of channel rhodopsin transfected cells [14], no current study employs optogenetic techniques in microfluidic cardiac models.

To perform more relevant studies specially designed microfluidic platforms were developed in the last years to provide a highly controllable biomimetic microenvironment and to model physiological functions of tissues [7]. In this framework, a matrix-free cardiac model was proposed by Mathur et al. [77], whose peculiarity was the possibility to tailor the diffusion of soluble molecules to the cardiac tissue constructs through microchannels, mimicking the human vasculature function. However, this platform did not allow to administer relevant cellular stimulation promoting the tissue formation. Conversely, Marsano et al. developed a microfluidic system gathering key biological and physical cues of the native myocardium, providing a 3D model to possibly predict hypertrophic cardiac phenotype changes by biochemical and mechanical co-stimulation [18]. A similar approach could have a huge impact in the field of muscle developmental biology, despite no such strategy yet being applied for the study of skeletal muscle.

Another important aspect which should be taken into account is the interaction between different cellular populations in a tissue, such as muscle cells and neurons. Several microfluidic platforms were designed to analyze formation, maintenance and disruption of engineered NMJs [39,40]. Even though NMJs represent a characteristic feature of the skeletal muscle, similar systems could be exploited to investigate the sympathetic neuron innervation of the heart, as recently reported by Takeuchi and colleagues [52].

From a reverse engineering point of view, it is important to understand which are the most critical features to fully recapitulate the functionality of a skeletal/cardiac tissue unit. In this context, we have highlighted that skeletal and cardiac muscles are characterized by unique properties, which have been successfully investigated by specific microfluidic models analyzing cell recruitment, cell-cell interactions or the effect of biophysical stimulations on the engineered tissue. Noteworthy, most of these models could be easily tailored for the study of both skeletal and cardiac muscle, by tuning specific parameters (e.g., stimulation pattern, force read-out, tissue specific molecular gradient, cell source). The peculiar design features and the optimization of microfluidic models, originally developed for the highly specific study of myoblasts or cardiomyocytes, could lead to a general progress of our knowledge of the pathophysiology of both skeletal and cardiac muscle.

## Figures and Tables

**Table 1:** Structural and functional comparison between key features of skeletal and cardiac muscle.

*Behavior*	*Skeletal*	*Cardiac*
*Gap junctions*	No	Yes
*Contraction regulation*	Voluntary	Involuntary
*Source of Ca++*	Sarcoplasmic reticulum	Sarcoplasmic reticulum and extracellular fluid
*Pacemaker*	No	Yes
*Electrical stimulation*	Nervous system (excitation)	Pacemaker (excitation). Nervous system (beating frequency modulation)
*Cell characteristic*	Long and cylindrical shape, multinucleate cell body with striation	Long and cylindrical (rod-shaped) cells, uni (fetal stage) or bi-nucleated (terminally differentiated), cell body with striation
